# Bidirectional Drive with Inhibited Hysteresis for Piezoelectric Actuators

**DOI:** 10.3390/s22041546

**Published:** 2022-02-17

**Authors:** Weiqing Huang, Junkai Lian, Dawei An, Mingyang Chen, Yinfeng Lei

**Affiliations:** School of Mechanical and Electrical Engineering, Guangzhou University, Guangzhou 510006, China; meehuangweiqing@gzhu.edu.cn (W.H.); lianjunkai@e.gzhu.edu.cn (J.L.); mingyangchen@e.gzhu.edu.cn (M.C.); leiyinfeng@e.gzhu.edu.cn (Y.L.)

**Keywords:** piezoelectric actuator, bidirectional drive, nonlinear displacement, precision drive

## Abstract

Piezoelectric actuators with a flexible displacement amplification structure are widely used in the fields of precision driving and positioning. The displacement curve of conventional piezoelectric actuators is asymmetrical and non-linear, which leads to large non-linear errors and reduced positioning accuracy of these piezoelectric actuators. In this paper, a bidirectional active drive piezoelectric actuator is proposed, which suppresses the hysteresis phenomenon to a certain extent and reduces the non-linear error. Based on the deformation theory of the beam, a theoretical model of the rhombus mechanism was established, and the key parameters affecting the drive performance were analyzed. Then, the static and dynamic characteristics of series piezoelectric actuators were analyzed by the finite element method. A prototype was manufactured and the output performance was tested. The results show that the actuator can achieve a bidirectional symmetric output of amplification displacement, with a maximum value of 91.45 μm and a resolution of 35 nm. In addition, compared with the hysteresis loop of the piezoelectric stack, the nonlinear error is reduced by 62.94%.

## 1. Introduction

High-precision positioning technology is one of the key technologies in ultra-precision machining, microelectronics, biological science, aerospace, and other fields [1,2,3]. In some precision positioning tasks, the actuator needs to achieve sub-nanometer resolution and a stroke of hundreds of microns to drive operational objects [4,5,6]. For example, in optical image stabilization, the position error of the lens is caused by external random vibration, and the clear imaging is realized by compensating the displacement error by the actuator [7,8]. As a key component of the micro-nano positioning system, the displacement output characteristics of the actuator have a great influence on the quality of precision positioning [9,10,11]. Therefore, it is necessary to design a high-performance micro-nano actuator to meet the demand for high-precision positioning technology in the field of microdrives. 

Many types of actuators have been developed according to different driving elements, such as electrothermal drives [12,13], shape memory alloy drives [14,15,16], and piezoelectric (PZT) stack drives [17,18]. Among them, PZT actuators have been used to develop various micro-displacement actuators or driving platforms because of their advantages of a small volume, fast response, zero-gap, and a large thrust–weight ratio [19,20,21,22,23]. The driving stroke of the PZT stack is only about 0.1% of its length, and so a flexible mechanism with a displacement amplification function is usually designed to enhance the driving stroke of the PZT stack [24]. At present, based on the displacement amplification principle of a flexible mechanism, the maximum stroke and minimum resolution of the actuator are the desired performance parameters. Many kinds of actuators based on the PZT stack have been developed. Mikio Muraoka et al. proposed a compact array design of a mechanical amplifier for PZT actuators, with a displacement magnification of seven times and a resonance frequency of 400 Hz [25]. Tajdari, F. et al. proposed a thin flexure-based mechanism that is useful in applications with limited building space. The proposed mechanism converts the initial in-plane motion of two PZT stack actuators to an out-of-plane translational motion [26]. Lin et al. derived the analytical model of actuator displacement amplification and calculated the attenuation displacement of the PZT stack in the positioning system [27]. Although the push stroke of the traditional PZT actuator is driven by the PZT stack, the return stroke can only be realized by the elastic recovery of the flexible mechanism. The driving displacement curve of the actuator presents an asymmetric and nonlinear complex shape, resulting in poor positioning accuracy of the push rod.

Although the displacement amplification mechanism enhances the driving stroke of the PZT stack, it also amplifies the hysteresis of the PZT stack. The actuator drive displacement curve exhibits a compound shape of asymmetry and nonlinearity, resulting in the poor positioning accuracy of the actuator. In the present stage of the research work, controllers are often used to compensate for the actuator error, and the latest hysteresis control strategies are reviewed in the literature [28]. Hysteresis of PZT stacks can also be suppressed using a charge drive; Liang Huang et al. proposed a switched capacitor charge pump to reduce hysteresis and linearize the motion of PZT actuators [29]. In addition, in the previous study, a bridge displacement amplification mechanism was optimized [30], which weakened the effect of hysteresis on the positioning accuracy of the actuator to some extent.

Aiming at the problem that the driving displacement curve of the traditional actuator presents an asymmetric and nonlinear complex shape, resulting in the low positioning accuracy of the actuator, a PZT actuator with a bidirectional symmetrical drive was proposed in this paper. Both push and return stroke can be driven actively, which overcomes the disadvantage that the traditional actuator can only rely on the elasticity of the structure to complete the return stroke, and improves the positioning accuracy of the actuator. Theoretical modeling of the actuator was carried out, and the structural parameters affecting the maximum stroke were analyzed. The maximum stroke, maximum stress point, and resonance frequency of the actuator were analyzed by means of the finite element method (FEM). An experimental test platform was established to test the maximum displacement, resolution, hysteresis, coupling displacement, response speed, and resonance frequency of the actuator.

## 2. Design of Series Structure

**Figure 1:**
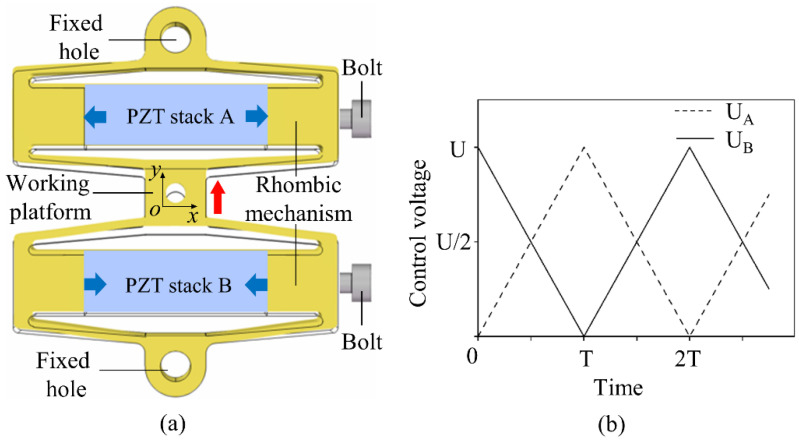
(**a**) The series rhombus PZT actuator. (**b**) Triangular wave control voltage.

There are various types of displacement amplifiers already in engineering applications, such as those with a lever mechanism [31], a Scott–Russell mechanism [32], and a pantograph mechanism [33], etc. Compared with other types of mechanisms, the rhombus-shaped displacement amplification mechanism is centrally symmetrical in shape and adopts the triangular displacement amplification principle, so it has both a large amplification ratio and a compact structure. The PZT stack can only bear compressive stress but not tensile stress, resulting in the different driving forces of the rhombus mechanism when reciprocating output displacement. Therefore, the displacement output curve of the rhombus mechanism shows an asymmetric complex shape. A series of rhombus mechanisms in a PZT actuator was proposed to realize the bidirectional active symmetric output of the actuator and suppress the hysteresis phenomenon. As shown in Figure 1a, the main structure of the actuator consists of a working platform and two rhombus mechanisms in series. The fixed holes are distributed on the outside of the rhombus mechanism, and the PZT stack is installed in the center of the rhombus mechanism. The bolts on the side can be adjusted to apply appropriate preload to the PZT stack to ensure that the PZT stack will not lose stability and fall off during operation. Table 1 lists the specific parameters of the PZT stacks used. 

Figure 1b shows the driving signals $U_{A}$ and $U_{B}$ of the PZT actuator, which are two triangular wave signals with 180° phase difference. PZT stack A is controlled by the driving signal $U_{A}$, and PZT stack B is controlled by the driving signal $U_{B}$. At 0–T, the voltage $U_{A}$ increases and PZT stack A elongates, pushing the external rhombus mechanism to deform and contract in the y direction, causing the working platform to move in the +y direction. At the same time, the voltage $U_{B}$ decreases, PZT stack B contracts, and its external rhombus mechanism elongates in the y direction, causing the working platform to move in the +y direction. In this process, the working platform moves in the +y direction, as shown by the red arrow in Figure 1a. At T–2T, the difference is that $U_{A}$ reduces while $U_{B}$ rises, and the working platform moves in the −y direction. Repeating the cycle 0–2T, the working platform will adopt a bidirectional symmetric drive.

## 3. Theoretical Model of Series Structure

**Figure 2:**
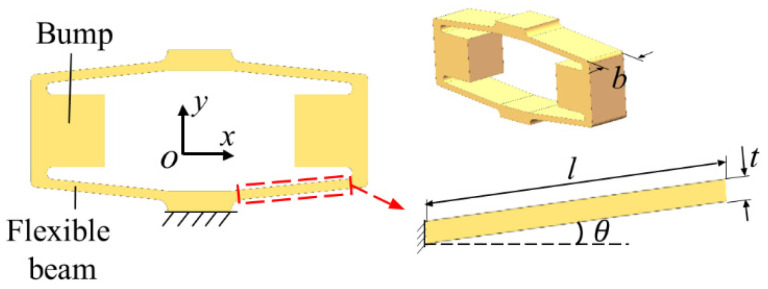
The diagram of the rhombus mechanism.

The displacement magnification and structural stiffness determine the maximum output displacement and maximum output force of the rhombic actuator, which can best represent the driving performance of the actuator. Therefore, the theoretical model of the rhombus actuator is established to determine the key parameters that affect the driving performance of the actuator. As shown in Figure 2, four flexible beams are connected by bumps to form the rhombus mechanism. The flexible beam is the key component of the rhombus mechanism, and the structural parameters are marked in the diagram—the thickness *t*, length *l*, inclination angle *θ,* and width *b* of the beam.

### 3.1. Displacement Magnification

**Figure 3:**
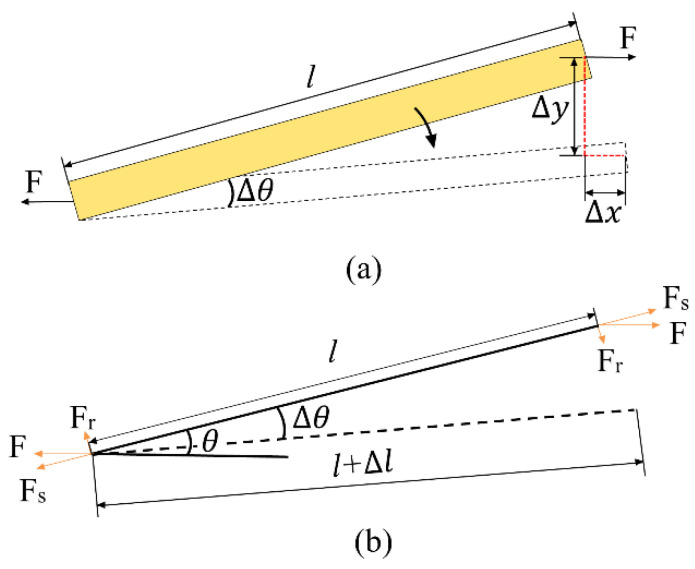
Force model of a flexible beam. (**a**) Structural deformation diagram. (**b**) Force analysis diagram.

Because the rhombus mechanism is centrosymmetric, the displacement characteristics of the rhombus mechanism can be studied by using its quarter model for analysis. Figure 3 depicts the force model of the flexible beam. During the operation of the rhombus mechanism, the flexible beam is slightly deformed and in the stage of linear elasticity. The PZT stack inputs a force 4*F* to the rhombus displacement amplification mechanism; a flexible beam subjected to force *F* produces an input displacement ∆*x* and output displacement ∆*y*, as well as a deflection angle ∆*θ*. As displayed in Figure 3b, *F* is decomposed into a tension $F_{s}$ along the flexible beam and a tension $F_{r}$ perpendicular to the flexible beam.

The relationship between force $F_{s}$ and elongation Δl can be expressed as follows:

(1) $$\Delta l=\frac{F_{s}}{k_{s}}$$

where $k_{s}$ is the tensile stiffness of the flexible beam.

(2) $$k_{s}=\frac{E\cdot b\cdot t}{l}$$

where *E* is the elastic modulus of the material, and *b*, *t*, and *l* are the width, thickness, and length of the flexible beam, respectively.

Due to the rotation of $F_{r}$ at both ends of the flexible beam, the flexible beam produces a rotation angle of ∆*θ*, and the moment balance equation is as follows:

(3) $$F_{r}l=2M=2k_{r}\Delta \theta$$

where $k_{r}$ is the rotational stiffness of the flexible beam and *M* is the bending moment at one end of the flexible beam.

(4) $$k_{r}=\frac{Ebt^{3}}{6l}$$

The displacement of Δx at the end of the flexible beam is caused by the force *F*, and the work done by the force *F* is expressed as follows:

(5) $$F\cdot \Delta x=F_{s}\cdot \Delta l+F_{r}\cdot l\cdot \Delta \theta$$

Substituting Equations (1)–(4) into (5), the displacement of Δx caused by the force *F* is obtained as follows:

(6) $$\Delta x=\frac{F\cdot \left(t^{2}\cdot \cos^{2}\theta \cdot l+12\cdot l^{3}\cdot \sin^{2}\theta \right)}{E\cdot b\cdot t^{3}}$$

**Figure 4:**
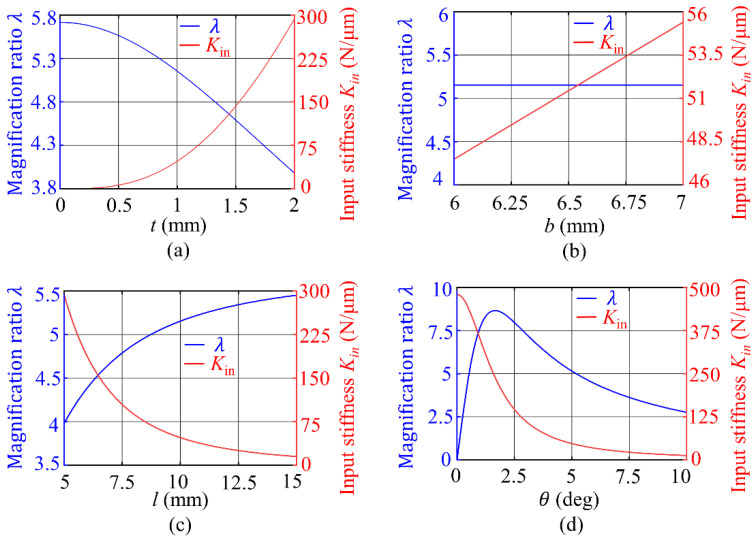
Influences of parameters on magnification and input stiffness. (**a**) Thickness. (**b**) Width. (**c**) Length. (**d**) Inclination angle.

According to the geometric relation in Figure 4a, the output displacement ∆*y* of the flexible beam is related to the deflection angle ∆*θ*, and the relation is expressed as follows

(7) $$\Delta y=\Delta \theta l\cos\theta =\frac{6F\sin\theta \cos\theta l^{3}}{Ebt^{3}}$$

The displacement magnification λ of the series structure can be expressed as follows:

(8) $$\lambda =\frac{\Delta y}{\Delta x}=\frac{3\cdot l^{2}\cdot \sin2\theta }{t^{2}\cdot \cos^{2}\theta +12\cdot l^{2}\cdot \sin^{2}\theta }$$

### 3.2. Maximum Drive Stroke of Series Structure

Through the analysis and calculation in the previous section, the expression of the input displacement of the flexible beam is obtained, and the input stiffness $K_{in}$ of the series structure can be expressed as:

(9) $$K_{in}=\frac{4\cdot F}{2\Delta x}=\frac{2\cdot E\cdot b\cdot t^{3}}{t^{2}\cdot \cos^{2}\theta \cdot l+12\cdot l^{3}\cdot \sin^{2}\theta }$$

Due to the influence of the input stiffness $K_{in}$ of the series structure, the relationship between the actual driving stroke Δx of the PZT stack and the driving stroke $x_{n}$ without load is as follows:

(10) $$\Delta x=\left(\frac{K_{PZT}}{K_{PZT}+K_{in}}\right)\cdot x_{n}$$

where $K_{PZT}$ is the stiffness of the PZT stack.

The theoretical calculation of the maximum displacement that can be output by the series structure is as follows:

(11) $$\Delta y_{max}=\lambda \cdot \left(\frac{K_{PZT}}{K_{PZT}+K_{in}}\right)\cdot x_{n_{max}}$$

where $x_{n_{max}}$ is the maximum driving stroke of the PZT stack without load. Experimental tests yielded the maximum unloaded displacement of the PZT stack $x_{n_{max}}=20.044\;\mu m$.

### 3.3. Analysis of Structural Parameters

According to Equation (11), a larger displacement magnification and a smaller input stiffness can cause the rhombus mechanism to obtain a larger output displacement. According to Equations (8) and (9), the structural parameters *t*, *l*, *b*, and *θ* determine the displacement amplification factor *λ* and input stiffness $K_{in}$ of the rhombus mechanism. The parameters of the series structure are analyzed by taking the initial values *t* = 1 mm, *l* = 10 mm, *b* = 6 mm, and *θ* = 5°. With the increase in flexible beam thickness, the displacement magnification decreases but the input stiffness increases, as depicted in Figure 4a. Therefore, the thickness of the flexible beam should be taken as the minimum possible. Considering the process level and practicability, *t* = 0.8 mm should be taken.

The flexible beam width has almost no effect on the displacement magnification, whereas the input stiffness is almost proportional to the flexible beam width, as shown in Figure 4b. Therefore, the width of the flexible beam should be equal to the width of the PZT stack (6 mm), which can be set as *b* = 6 mm.

The displacement magnification increases with the increase in flexible beam thickness, but the input stiffness decreases with the increase in flexible beam thickness, as presented in Figure 4c. The flexible beam thickness is determined as *l* = 12 mm.

The displacement magnification of the rhombus mechanism reaches its maximum value when the inclination angle *θ* is equal to 1.5°, as displayed in Figure 4d. When the angle is smaller than 1.5°, the displacement magnification increases with the angle, and when the angle is larger than 1.5°, the displacement magnification decreases with the angle. The input stiffness decreases with an increase in the inclination angle. To reduce the influence of machining accuracy on the displacement output performance of the actuator, the inclination angle should be the area with a relatively gentle curve change, taking *θ* = 5°.

**Figure 5:**
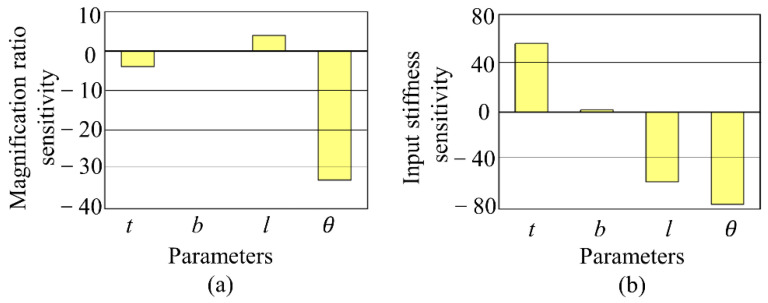
Sensitivity analyses results. (**a**) magnification sensitivity. (**b**) input stiffness sensitivity.

Using sensitivity analysis, the variation of the maximum driving stroke can be studied when the structural parameters are inaccurate or change, and high sensitivity structural parameters should be the primary guarantee of machining accuracy. From Equation (11), the maximum driving stroke $\Delta y_{max}$ is related to the displacement amplification *λ* and the input stiffness $K_{in}$. Based on the parameter sensitivity analysis of displacement amplification *λ* and input stiffness $K_{in}$ in Figure 4, the change rate of displacement amplification *λ* and input stiffness $K_{in}$ are calculated, respectively, when the structural parameters vary by 10%, and the results are shown in Figure 5. Compared with other parameters, the displacement amplification and input stiffness of the series structure are more sensitive to the change in the inclination angle *θ* of the flexible beam, and the accuracy of the inclination angle of the flexible beam should be ensured first when the prototype is processed.

The main parameters of the series structure PZT actuator and the physical and mechanical parameters of 45# steel are listed in Table 2. The displacement magnification ratio is calculated as λ=5.54, the input stiffness is calculated as $K_{in}=14.88\;N/\mu m$ and the maximum drive stroke is calculated as $\Delta y_{max}=88.98\;\mu m$.

## 4. Discussion Results of Finite Element Method

**Figure 6:**
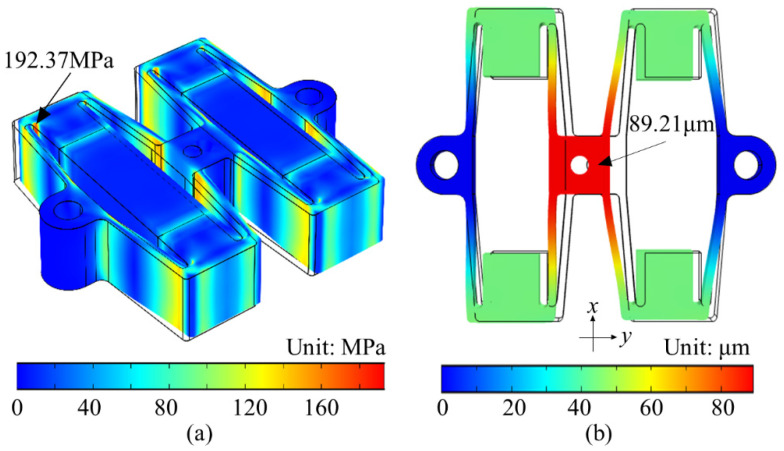
Static analysis of PZT actuator. (**a**) Stress analysis. (**b**) Displacement analysis.

Static and dynamic analysis of the actuator was performed by COMSOL Multiphysics software to verify the analytical model. The material of the tandem structure is set to 45 steel, and the material of the PZT stack uses PZT-5H, and the two fixing holes were set to fixed constraints. When a voltage is applied to the PZT stack and its output displacement is 16.03 μm (equivalent to the maximum driving stroke of the PZT stack after the action of input stiffness), the deformation of each part is presented in Figure 6. The displacement output direction of the target working platform meets the requirements.

The stress distribution of the PZT actuator is shown in Figure 6a. The maximum stress is 192.37 MPa generated at the connection between the flexible beam and the bump, which is far less than the yield strength (355 MPa) of the material. The actuator is always in the linear elastic stage during the actuation process. The displacement distribution of the PZT actuator in the *y*-direction is displayed in Figure 6b. The maximum driving stroke of the PZT actuator is 89.21 μm, with a magnification of 5.68, and the errors of 0.26% and 2.53% with the theoretical model, respectively.

**Figure 7:**
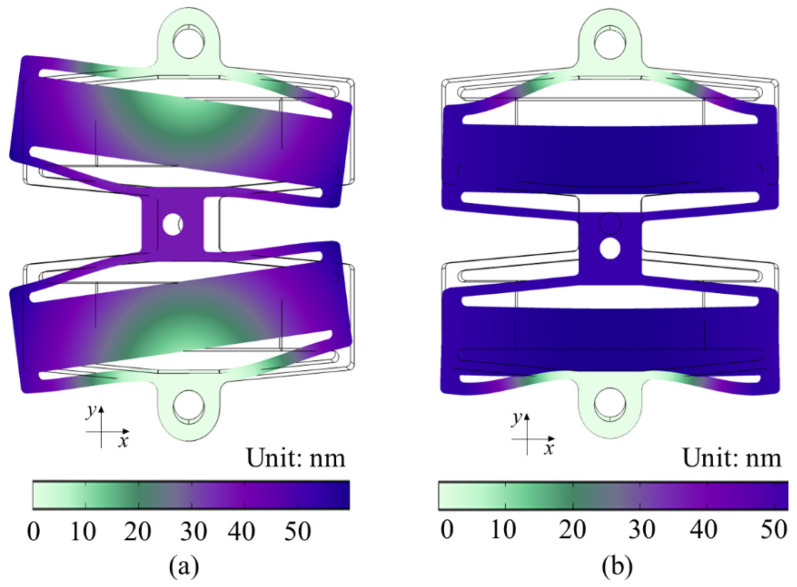
First two-mode shapes. (**a**) First resonance frequency: 1455.4 Hz. (**b**) Second resonance frequency: 1615 Hz.

Modal analysis was utilized to examine the dynamic performance of the series rhombus PZT actuator. Figure 7 shows the modal analysis results with the first two-mode shapes and corresponding natural frequencies. As displayed in Figure 7a, the first mode shape of the actuator is the rotation of two rhombus mechanisms around the fixed hole on the outside, and the working platform in the center moves in the *x*-direction, with a natural frequency of 1455.4 Hz. As displayed in Figure 7b, the second mode shape of the actuator is the overall movement of the actuator in the y-direction, and its natural frequency is 1615 Hz. The results of frequency domain analysis show that the series rhombus PZT actuator has a high first two resonance frequencies and can meet the needs of precision positioning at a low frequency.

## 5. Experiments

**Figure 8:**
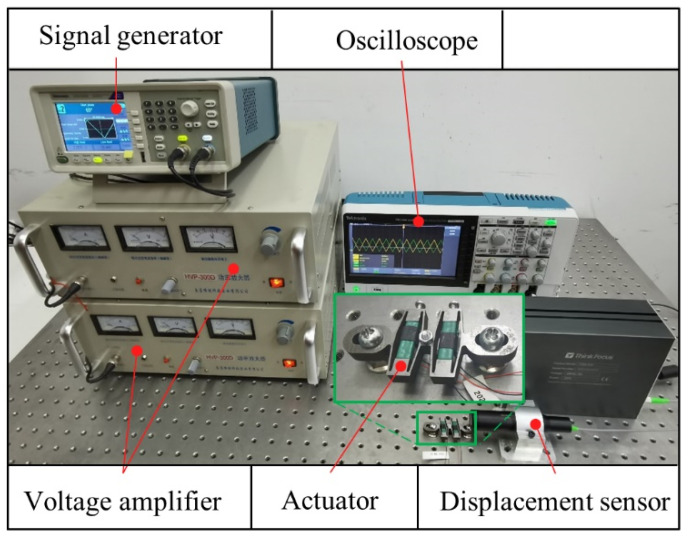
Experimental setup.

The prototype of the bidirectional active drive PZT actuator was integrally manufactured by wire cutting technology, with a size of 38 × 32 × 6 mm^3^ and a weight of 30.1 g. The size of the PZT stack was 6 × 6 × 18 mm^3^ and the maximum free extension stroke was 20.044 μm. The experimental platform consisted of a series structure PZT actuator, a signal generator (Tektronix AFG1022, Tektronix Corporation, Beaverton, OR, USA), a power amplifier (Nanjing Foneng HVP-300D, Nanjing, China), an oscilloscope (Tektronix TBS2000, Tektronix Corporation, Beaverton, OR, USA), a spectral confocal displacement sensor (Think Focus CDS-500, THINKFOCUS, Shanghai, China), and a computer. Two voltage signals were generated by the signal generator, amplified by the power amplifier, and applied to the PZT stack. The oscilloscope was used to detect the waveform and amplitude of the voltage, and the driving displacement of the actuator was detected by a displacement sensor. The displacement data were transmitted back to the computer. All devices were placed on a vibration isolation table, as displayed in Figure 8.

**Figure 9:**
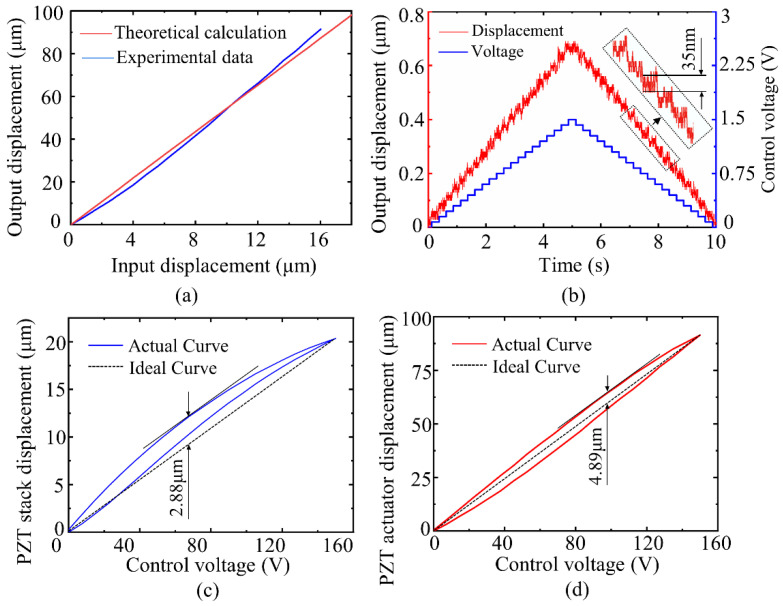
(**a**) Displacement magnification test. (**b**) Resolution of the actuator. (**c**) Hysteresis loop of the PZT stack. (**d**) Hysteresis loop of the actuator.

Firstly, the maximum output displacement of the actuator was tested to determine the maximum stroke and displacement magnification of the actuator. A triangle wave signal with a peak-to-peak value of 150 V was applied to the PZT stack, and the experimental results are shown in Figure 9a. The maximum output displacement of the actuator was 91.45 μm, and the displacement magnification was 5.71 times, both of which were larger than the theoretical calculation values, and the errors were 2.7% and 4.55%, respectively. There are two reasons for the discrepancy between the measured and calculated values. The first is the error of processing and manufacturing. Secondly, in theoretical modeling, only the deformation of the flexible beam is considered while other parts are ignored, resulting in the deviation of the theoretical model.

The minimum step displacement determined the positioning accuracy of the actuator. A step voltage was applied to the PZT stack to test the step accuracy of the actuator. At a step voltage of 0.075 V per stage, the displacement curve of the PZT actuator is presented in Figure 9b, which is affected by the noise of the voltage signal, and the step displacement of the actuator starts to overlap with the noise. Under the open-loop control, the displacement resolution of the actuator reaches 35 nm.

The shape of the hysteresis loop determines the difficulty in controlling the complete linear displacement of the actuator by the algorithm. A triangular wave voltage with an amplitude of 150 V and a frequency of 1 Hz is applied to the PZT stack and the actuator, respectively, and the displacement curve of one cycle is measured and plotted to obtain the two plots shown in Figure 9c,d. The maximum displacement deviation is 2.88 µm and the maximum nonlinear error is 14.41% for the PZT stack, while the maximum displacement deviation is 4.89 µm and the maximum nonlinear error is 5.34% for the proposed actuator. The results show that the hysteresis loop is symmetrical and the maximum nonlinear error is improved.

**Figure 10:**
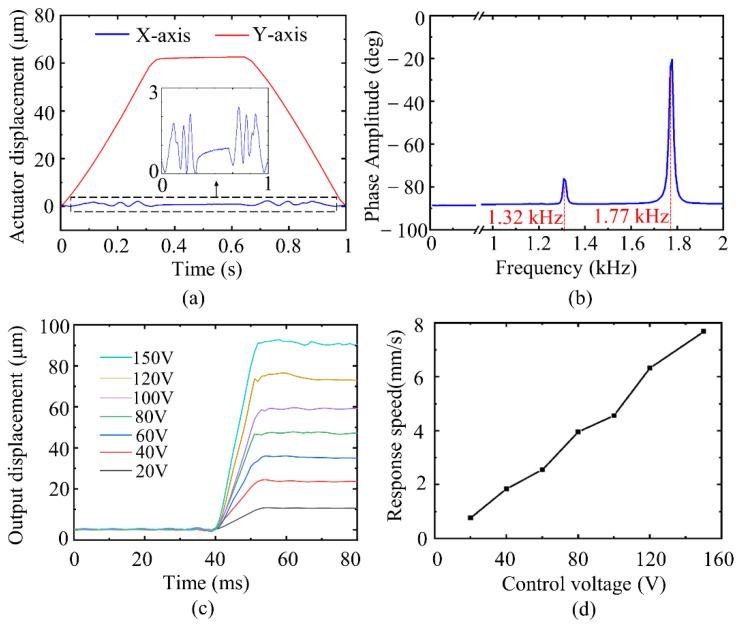
(**a**) coupling displacement. (**b**) frequency response. (**c**) response times. (**d**) response speed.

The trapezoidal voltage signal with a slope of 300 V/s and the maximum voltage of 100 V was applied to the PZT stack. The driving stroke of the actuator is indicated in Figure 10a. The maximum displacement of the working platform in the y-direction was 62.56 μm, and the maximum coupling displacement in the *x*-direction was 2.33 μm. The coupling displacement of the working platform fluctuated greatly during the rising and falling stages of the voltage. The structure of the actuator was not perfectly symmetrical under actual machining conditions, resulting in different output forces of the PZT stack.

The dynamic characteristics of the actuator were tested by frequency response. A frequency swept sine wave with the amplitude of 1 V and frequency range of 10~2000 Hz was applied to the PZT stack to obtain the frequency response curve of the actuator, as displayed in Figure 10b. The first two resonance frequencies are 1.32 and 1.77 kHz, respectively, and the error between the results and FEM is 10.26% and 9.60%, respectively.

The output response speed of the actuator was tested by applying square wave signals of the same frequency and different amplitudes to the PZT stack. As seen from Figure 10c, the measured data were low-pass filtered to obtain the displacement curves of the working platform under the step voltage of 20–150 V. The response time of the actuator was kept in the range of 11–13 ms under different step voltages, and the response speed of the actuator was approximately linear with the control voltage, as shown in Figure 10d.

## 6. Conclusions

In this work, a bidirectional active drive PZT actuator has been developed to solve the problems of complex output displacement curve shapes and poor positioning accuracy of conventional actuators. The actuator can output a driving stroke of 91.45 μm and achieve a resolution of 35 nm. Firstly, the structure of the series PZT actuator was designed and the corresponding theoretical model was established to analyze the structural parameters that affect the maximum stroke of the actuator. The maximum stress, maximum stroke, displacement magnification, and resonance frequency of the actuator were calculated by FEM. The test results of the actuator prototype show that the output displacement curve of the actuator has a symmetrical shape.

The comparison between the proposed actuator and those of similar studies is listed in Table 3. It can be seen that the proposed actuator has advantages in terms of nonlinear error, resolution, and first-order resonant frequency. 

## Figures and Tables

**Table 1:** Parameters of PZT stacks.

Manufacturer	Model	PZT Ceramic Parameters				
		Material	Number	Thickness (μm)	Area (mm^2^)	PZT Constant (pm/V)
COREMORROW	Pst150/5 × 5 × 20	PLZT	200	100	25	+635

**Table 2:** Main parameters of the series structure PZT actuator.

**Parameters**	*t* (mm)	*l* (mm)	θ (deg)	*b* (mm)	*E* (GPa)	μ	*ρ* (kg/m^3^)	λ	*K_in_* (N/μm)	Δ*y_max_* (μm)
**Value**	0.8	12	5	6	206	0.269	7850	5.54	14.88	88.98

**Table 3:** Performance comparison of different actuators.

Reference	[5]	[34]	[35]	[30]	This Work
Dimension (mm^3^)	63 × 48 × 10	-	-	38 × 32 × 6	45 × 30 × 6
Maximum Stroke (μm)	+488	±720	+288.3	+129.41	+91.45
Resolution (nm)	50	-	50	-	35
Resonance frequency (Hz)	130	628	178	377.2	1320
Nonlinear error	11.8%	≈15%	10.63%	5.48%	5.34%

## Data Availability

Data supporting the findings of this study are contained within the article. Any further data required are available from the corresponding author upon request.
